# *CCND1* copy number increase and cyclin D1 expression in acral melanoma: a comparative study of fluorescence in situ hybridization and immunohistochemistry in a Chinese cohort

**DOI:** 10.1186/s13000-021-01116-0

**Published:** 2021-07-05

**Authors:** Jianying Liu, Wenjuan Yu, Fei Gao, Shuangshuang Qi, Juan Du, Xiaolong Ma, Yan Zhang, Jie Zheng, Jing Su

**Affiliations:** 1 Department of Pathology, School of Basic Medical Sciences, Third Hospital, Peking University Health Science Center, 38 Xueyuan Road, Beijing, 100191 China; 2 Department of Pathology, The Affiliated Hospital of Qingdao University, Qingdao, 266003 China

**Keywords:** *CCND1* (cyclin D1), Acral melanoma, Gene copy number increase, Protein expression

## Abstract

**Background:**

*CCND1* copy number increase is characteristic of acral melanoma and is useful in distinguishing benign and malignant acral melanocytic lesions. Increase of the gene copy number may result in protein overexpression. This raises the possibility that detection of high expression of cyclin D1 by immunohistochemistry (IHC) may be used as a surrogate for direct evaluation of increase in the *CCND1* gene copy number.

**Methods:**

We examined increases in *CCND1* copy number with fluorescence in situ hybridization (FISH), and examined cyclin D1 protein expression with IHC in 61 acral melanomas.

**Results:**

Using FISH, 29 acral melanomas (29/61, 47.5%) showed increase in the *CCND1* copy number, including 8 (8/61, 13.1%) which showed low-level increase in the *CCND1* copy number and 21 (21/61, 34.4%) with high-level increase in the *CCND1* copy number. By analysis of IHC, the median IHC score was 15% (range: 1–80%) in acral melanomas with no *CCND1* copy number alteration. In acral melanomas with low-level *CCND1* copy number increase, the median IHC score was 25% (range: 3–90%). In acral melanomas with high-level *CCND1* copy number increase, the median IHC score was 60% (range: 1–95%). Comparing FISH and IHC, cyclin D1 protein expression level has no corelation with the *CCND1* copy number in acral melanomas which have no *CCND1* copy number alteration and low-level *CCND1* copy number increase (*P* = 0.108). Cyclin D1 protein expression level correlated positively with *CCND1* copy number in acral melanomas with high-level *CCND1* copy number increase (*P* = 0.038). The sensitivity, specificity and positive predictive value of using cyclin D1 IHC to predict *CCND1* FISH result was 72.4, 62.5 and 63.6%. Increase in *CCND1* copy number was associated with Breslow thickness in invasive acral melanoma.

**Conclusion:**

High-level increase in the *CCND1* copy number can induce high cyclin D1 protein expression in acral melanomas. However low-level increase and normal *CCND1* copy number have no obvious correlation with protein expression. Cyclin D1 IHC cannot serve as a surrogate for *CCND1* FISH in acral melanomas.

## Introduction

Acral melanoma is a distinct subtype of melanoma that most commonly affects the Asian population and has worse survival than other cutaneous melanomas [1, 2]. Acral melanoma may be particularly difficult to distinguish from acral nevus by histopathology, and ancillary methods that help establish the diagnosis may be useful. The *CCND1* gene which is located on chromosome 11q13 is a proto-oncogene which is transcribed to protein cyclin D1, and cyclin D1 forms active complexes with CDK4/CDK6, resulting in phosphorylation of the retinoblastoma protein (Rb) which drives G1 to S phase [3]. Abnormalities of the *CCND1* gene are found in some malignant melanocytic tumors, and especially in acral melanoma [4, 5]. In acral melanoma, most *CCND1* abnormalities are characterized by an increase of the gene copy number, and *CCND1* copy number changes are not found in acral melanocytic nevi [5, 6]. A fluorescence in situ hybridization (FISH) panel including *CCND1* has proved to be an effective means of distinguishing benign and malignant melanocytic tumors, including acral melanocytic tumors [7–13].

Gene copy number increase in cancer-promoting driver gene in malignant cells may result in protein overexpression, such as in human epithelial growth receptor 2 (HER2) on chromosome 17 [14]. In breast cancer and gastric cancer, there is good correlation in HER2 gene copy number increase and protein overexpression, which allows use of immunohistochemistry (IHC) in these tumors as a method for preliminary screening before resorting to FISH [15, 16]. We wished to determine whether increases in *CCND1* gene copy number and cyclin D1 protein expression is correlated in acral melanoma. If so, IHC has potential to serve as a preliminary screening method which is both easier technically and more economical than FISH.

The aim of this study was to evaluate the consistency of *CCND1* copy number increase with cyclin D1 protein expression in acral melanomas, and to assess the potential role of cyclin D1 IHC serving as a preliminary screening method for *CCND1* FISH. For this purpose, we evaluated 61 acral melanomas for *CCND1* copy number alteration and cyclin D1 expression.

## Materials and methods

### Patients

A total of 61 successive and unselected cases of acral melanoma were collected from the Department of Pathology, School of Basic Medical Sciences, Third Hospital, Peking University Health Science Center from January 2013 to October 2018. In addition to these 61 acral melanomas, 26 benign acral melanocytic nevi were also collected and evaluated. All specimens were fixed in formalin and embedded in paraffin. Two pathologists (Jianying Liu and Jing Su) read these cases independently to confirm the diagnoses. This study was approved by the Research Ethics Committee, Peking University Health Science Centre, Beijing, China.

### Fluorescence in situ hybridization and signal measurement

*CCND1* FISH analysis was conducted as previously described using the Vysis Melanoma FISH Probe Kit purchased from Abbott Molecular Inc. (Des Plaines, IL, USA) [8]. After hybridization, FISH slides were screened at high magnification (× 100 objective with oil immersion). A total of 30 non-overlapping intact tumor nuclei were counted for each slide. The average copy number for the *CCND1* gene site was calculated.

When the average copy number for *CCND1* was ≥2.50, the tumor was considered to have an increase in *CCND1* copy number. When the average copy number of *CCND1* was ≥2.50 but <4.00, the tumor was classified as having a low-level increase in *CCND1* copy number; and when the average copy number of *CCND1* ≥ 4.00, the tumor was considered to have a high-level increase in *CCND1* copy number.

### Immunohistochemistry and evaluation of immunostaining

Cyclin D1 IHC was performed with a LEICA BOND-MAX system using Cyclin D1 Rabbit monoclonal antibody (Cell Marque, California, USA). The percentage of positive cells (nuclear staining) was scored by two pathologists (Jianying Liu and Jing Su) who were blinded to the FISH results. The average score generated by these two pathologists was used as the final IHC score.

### Statistical analysis

**Fig. 1:**
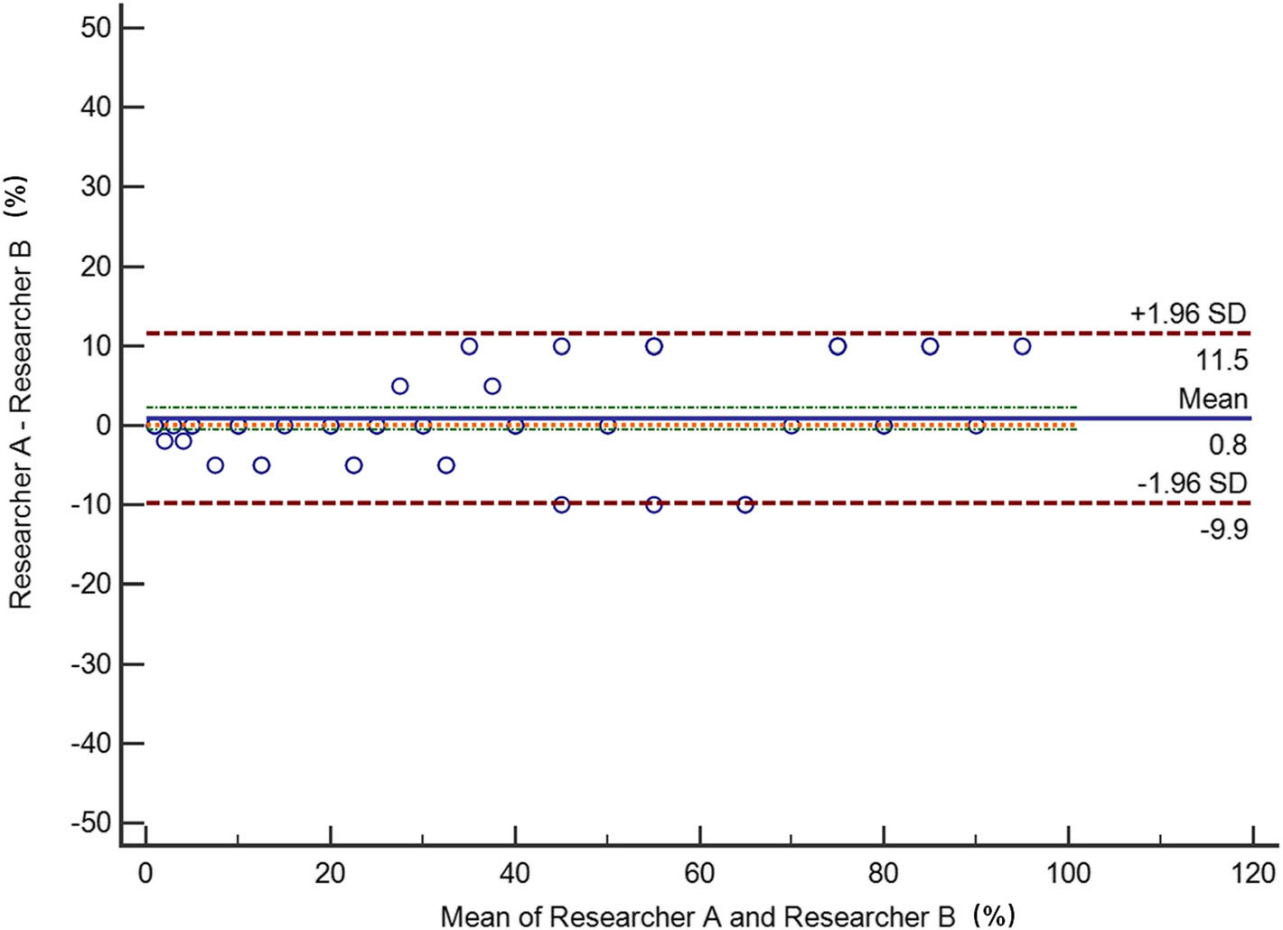
Bland Altman plot for the cyclin D1 IHC score by the two pathologists (Jianying Liu and Jing Su)

The intraclass correlation coefficient of the IHC scores for Jianying Liu and Jing Su was calculated. The intraclass correlation coefficient of the IHC scores generated by the two pathologists (Jianying Liu and Jing Su) was above 90%. The Bland Altman plot (Fig. 1) shows the difference mean between the two pathologists is 0.8%, the standard deviation (SD) is 5.4% and the range between difference mean ± 1.96SD is from − 9.9 to 11.5%. These imply that there is good agreement between the two pathologists. The average score for these two pathologists was used as the final IHC score.

The correlation between the *CCND1* gene copy number and cyclin D1 protein expression was evaluated with Spearman correlation. The most effective cut-off score for cyclin D1 IHC (percentage of positive cells) for predicting FISH results was calculated with ROC curves. The specificity, sensitivity, positive predictive value and negative predictive value of using cyclin D1 IHC scores to predict *CCND1* FISH results was calculated with binary logistic regression and ROC curve. The relationship of *CCND1* gene copy number alterations and patient gender, as well as tumor ulceration was assessed with the Pearson’s chi-square χ^2^ test. The relationship of the *CCND1* gene copy number alterations and other clinicopathologic parameters (patient age, Breslow thickness and Clark’s level) were assessed with the independent T test. The relationship of cyclin D1 expression status and patient gender, as well as tumor ulceration were assessed with nonparametric tests. The relationship of cyclin D1 expression status and other clinicopathologic parameters (patient age, Breslow thickness and Clark’s level) were assessed with Spearman correlation. All statistical data were calculated using IBM SPSS statistics 23 (USA). All *p* values were two-sided. *P* values < 0.05 were considered statistically significant.

## Results

### Clinicopathologic characteristics

The clinical and pathologic features of the 61 acral melanoma patients evaluated in this study are summarized in Table 1. Thirty-two melanoma patients were male and 29 were female (male-to-female ratio 1.1:1). The median patient age was 62 years with a range of 22 to 87 years. Histologic subtypes included acral lentiginous melanoma (43/61, 70.5%) and nodular melanoma (18/61, 29.5%). The mean Breslow thickness was 4.3 mm (range 0.5 mm to 30.0 mm). Ulceration was observed in 27 cases (27/61, 44.3%).

**Table 1:** Clinical and pathological features of the acral melanoma patients (61 cases)

Feature	Number of patients (%)
Gender	
Male	32 (52.5%)
Female	29 (47.5%)
Age at surgery (years)	
Median age	62
Range	(22–87)
Site	
Foot	39 (63.9%)
Hand	17 (27.9%)
Nail	5 (8.2%)
Histological subtype	
Acral-lentigous melanoma	43 (70.5%)
Nodular melanoma	18 (29.5%)
Breslow thickness	
≤ 1 mm	11 (18.0%)
> 1.0 mm–2.0 mm	17 (27.9%)
> 2.0 mm–4.0 mm	16 (26.2%)
> 4.0 mm	17 (27.9%)
Mean tumor thickness (mm)	4.3
Median tumor thickness (mm)	2.5
Ulceration	
Yes	27 (44.3%)
No	34 (55.7%)
Clark’s level	
I	7 (11.5%)
II	3 (4.9%)
III	5 (8.2%)
IV	37 (60.7%)
V	9 (14.8%)

A total of 26 benign acral melanocytic nevi from 12 male and 14 female patients of ages 5 to 58 years (median age 29) were evaluated. These nevi were all of conventional type, and included 15 compound nevi and 11 intradermal nevi. The sites included palm (5/26, 19.2%) and sole (21/26, 80.8%).

### CCND1 copy number alteration in acral melanomas

**Fig. 2:**
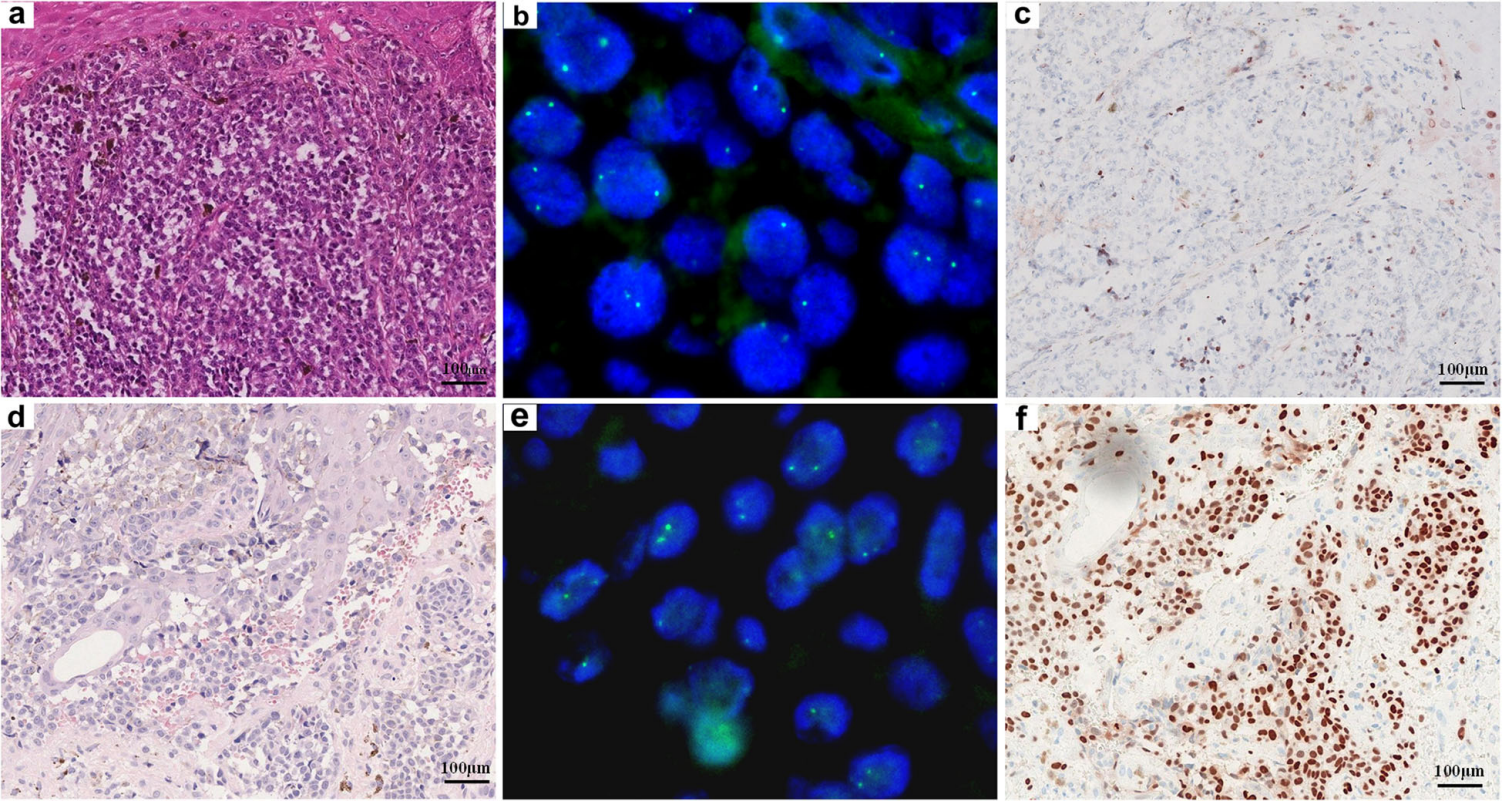
Acral melanomas with normal *CCND1* copy number may show either low- or high-expression of cyclin D1 protein. An acral melanoma on the thumb of a 57-year-old male with normal *CCND1* copy number shows low expression of cyclin D1 protein (**a**, HE 100x; **b**, *CCND1* copy number by FISH; **c**, cyclin D1 expression by IHC). An acral melanoma on the hand of an 80-year-old female with normal *CCND1* gene copy number shows high expression of cyclin D1 protein (**d**, HE 100x; **e**, *CCND1* copy number by FISH; f, cyclin D1 expression by IHC)

**Fig. 3:**
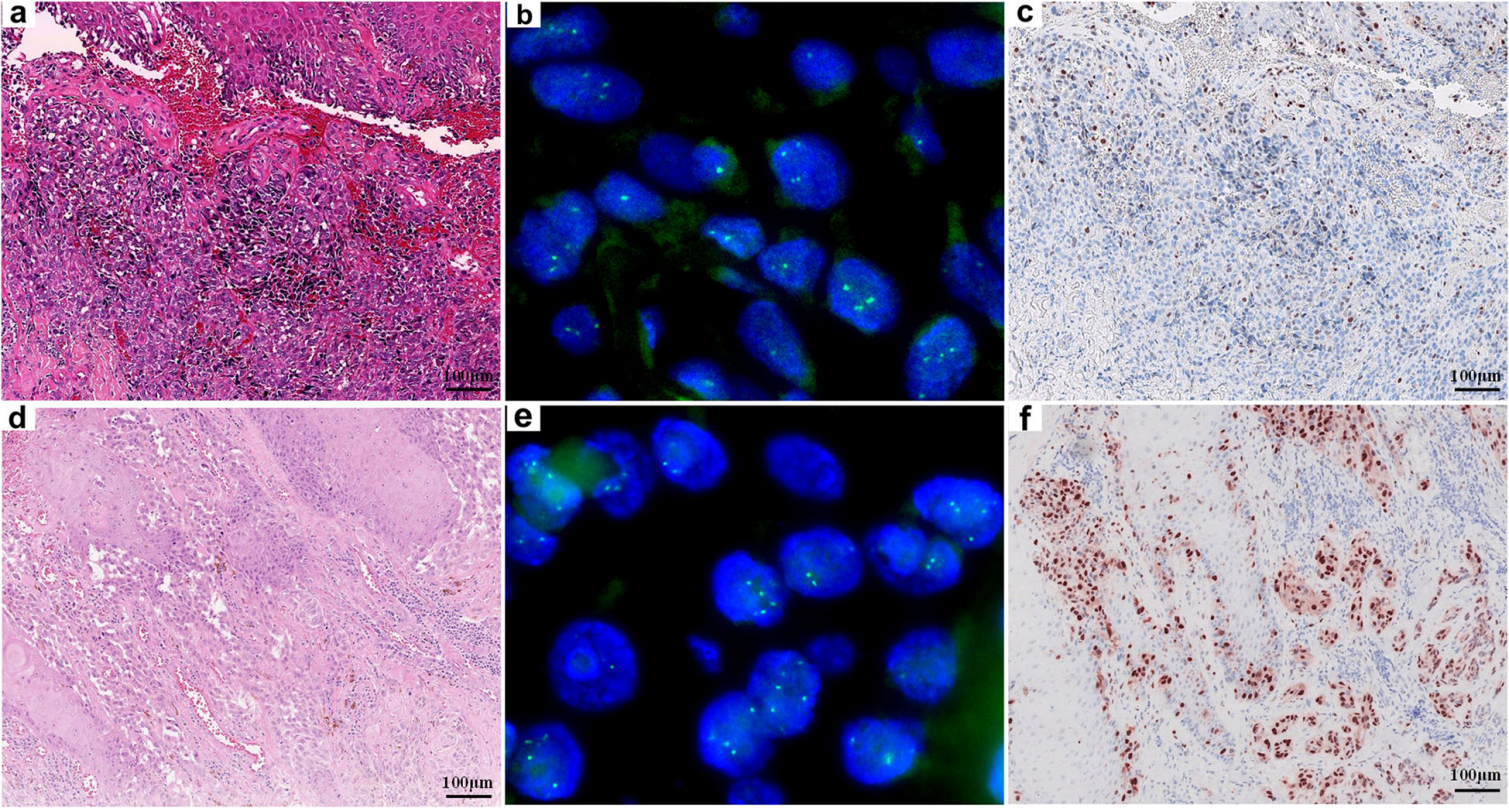
Acral melanomas with low-level *CCND1* copy number increase may show either low- or high-expression of cyclin D1 protein. An acral melanoma on the heel of a 74-year-old female with low-level *CCND1* gene copy number increase shows low expression of cyclin D1 protein (**a**, HE 100x; **b**, *CCND1* copy number by FISH; **c**, cyclin D1 expression by IHC). An acral melanoma on the sole of a 53-year-old male with low-level *CCND1* copy number increase shows high expression of cyclin D1 (**d**, HE 100x; **e**, *CCND1* copy number by FISH; f, cyclin D1 expression by IHC)

**Fig. 4:**
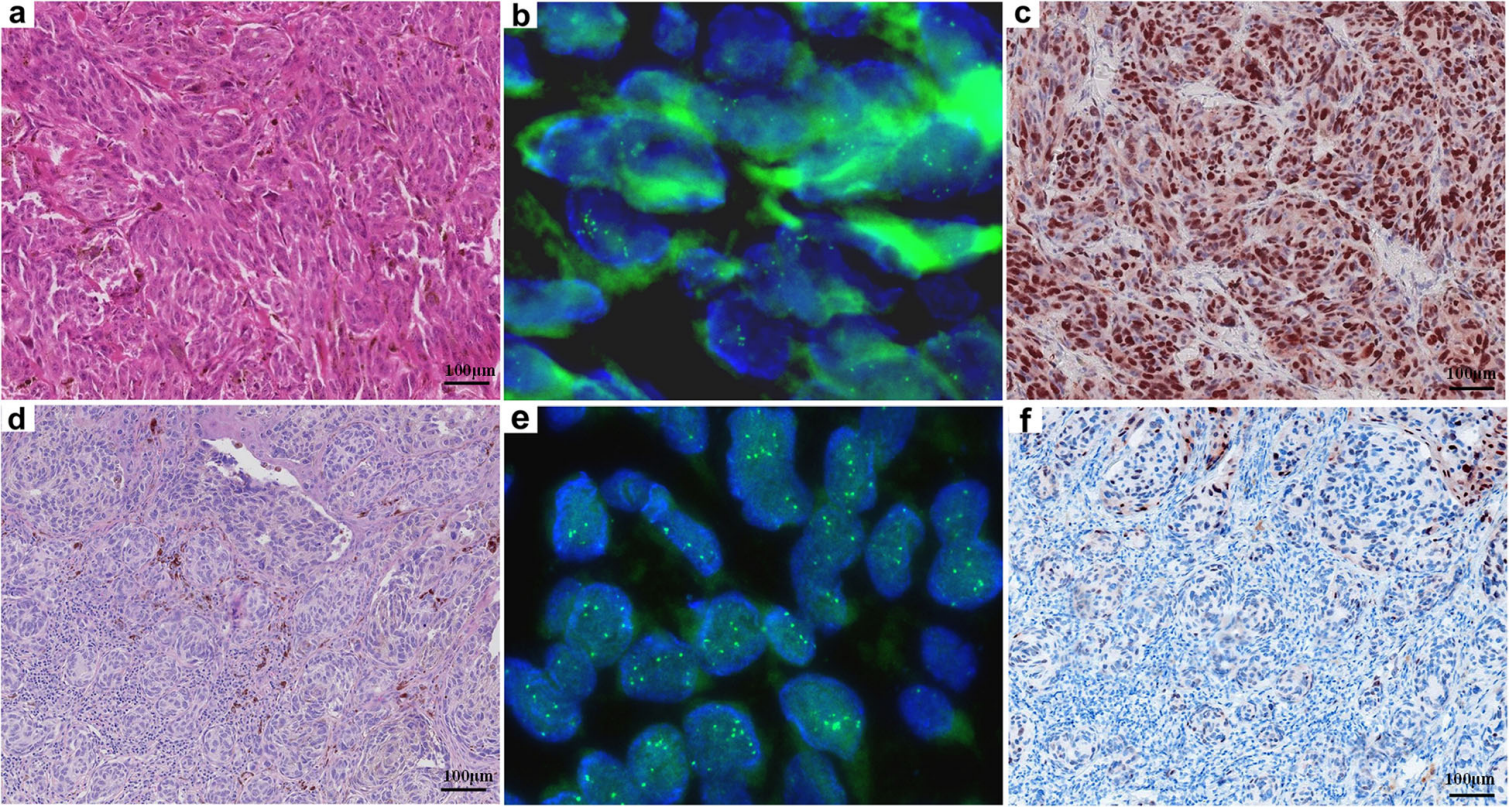
Acral melanomas with high-level *CCND1* copy number increase shows high cyclin D1 expression in most cases, but can also show low cyclin D1 expression in a small number of cases. An acral melanoma on the foot of a 49-year-old female with high-level *CCND1* copy number increase shows high expression of cyclin D1 (**a**, HE 100x; **b**, *CCND1* copy number by FISH; **c**, cyclin D1 expression by IHC). An acral melanoma on the foot of a 47-year-old male with high-level *CCND1* copy number increase shows low expression of cyclin D1 (**d**, HE 100x; **e**, *CCND1* copy number by FISH; **f**, cyclin D1 expression by IHC)

Thirty-two acral melanomas (52.5%, 32/61) showed no *CCND1* copy number alterations (Fig. 2b and e). Twenty-nine acral melanomas (47.5%, 29/61) showed increased *CCND1* copy number. Eight of these (8/61, 13.1%) showed low-level copy number increase (average copy number ≥ 2.5 and < 4.0, Figs. 3b and e) and 21 (21/61, 34.4%) showed high-level copy number increase (average copy number ≥ 4.0, Fig. 4b and e).

### Cyclin D1 expression in acral melanomas

Nuclear cyclin D1 expression was found in all 61 acral melanomas using IHC. The median IHC score in acral melanoma was 30% (range: 1–95%). In acral melanomas with no *CCND1* copy number alteration, the median IHC score was 15% (range: 1–80%) (Fig. 2c and f). In acral melanomas with low-level *CCND1* copy number increase, the median IHC score was 25% (range: 3–90%) (Fig. 3c and f). In acral melanomas with high-level *CCND1* copy number increase, the median IHC score was 60% (range: 1–95%) (Fig. 4c and f). The median IHC score for acral nevi was 10% (range: 1–30%).

### Comparison of CCND1 copy number alteration and cyclin D1 protein expression in acral melanomas

**Fig. 5:**
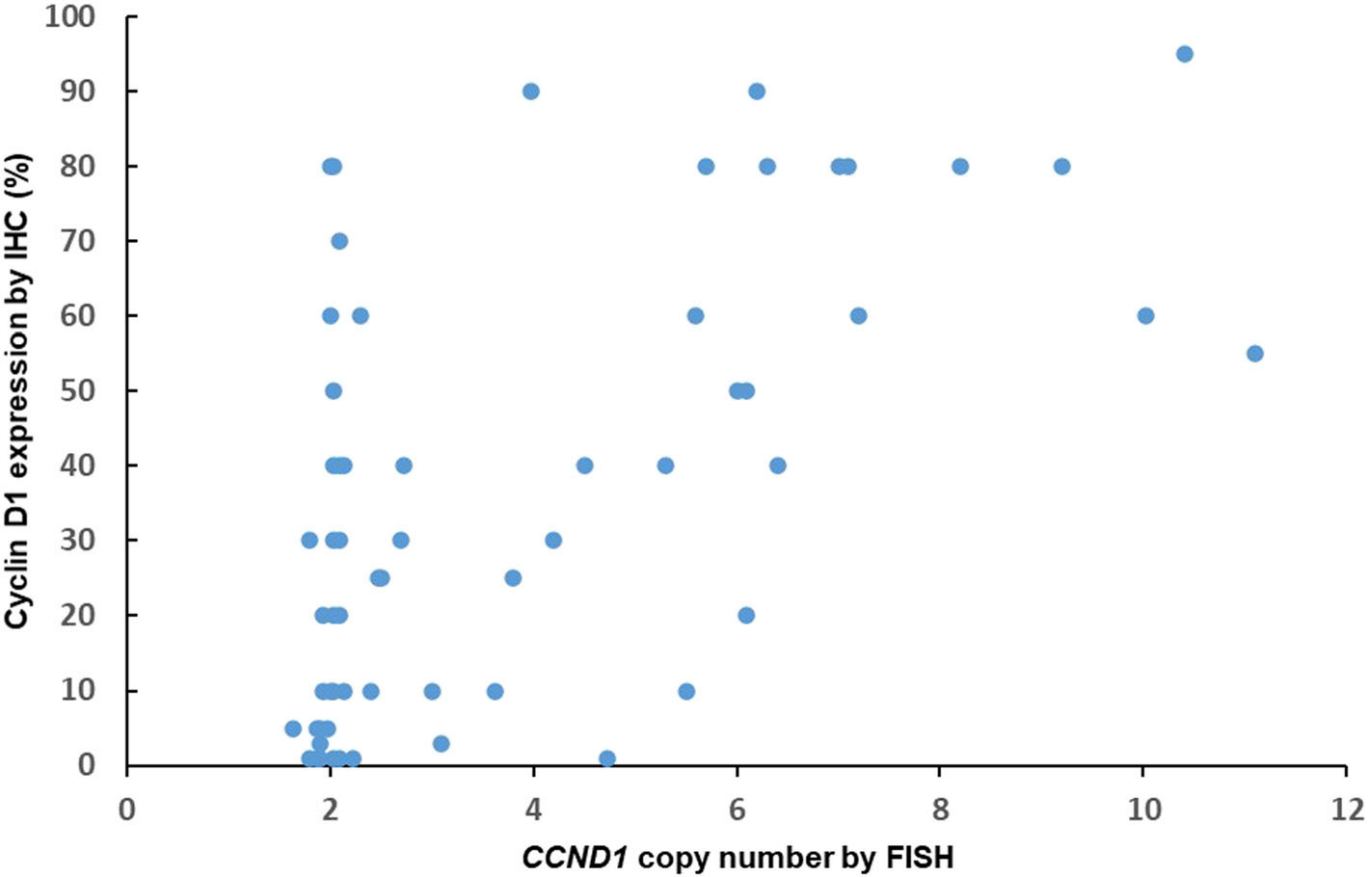
The scatter plot shows the correlation of the *CCND1* copy number by FISH and the cyclin D1 expression by IHC

The correlation of *CCND1* gene copy number and cyclin D1 protein expression is shown in Fig. 5. The cyclin D1 protein expression level has no corelation with *CCND1* copy number in acral melanomas with no *CCND1* copy number alteration and low-level copy number increase (*P* = 0.108). The cyclin D1 protein expression level correlates positively with the *CCND1* copy number in acral melanomas with high-level *CCND1* copy number increase (*P* = 0.038).

### Using cyclin D1 IHC score to predict CCND1 FISH result

Using ROC curves, we found that 27.5% is the most effective cyclin D1 IHC cut-off for predicting *CCND1* FISH results, with a sensitivity of 72.4% and a specificity of 62.5%. The positive predictive value is 63.6% and negative predictive value is 71.4%. The cyclin D1 IHC score does not predict *CCND1* copy number alterations properly.

### Correlation of FISH and IHC results with clinicopathologic characteristics

*CCND1* copy number increase is associated with the Breslow thickness (*P* = 0.043) in invasive acral melanomas. There were no *CCND1* copy number changes associated with other clinicopathologic parameters under evaluation, including patient age (*P* = 0.128), gender (*P* = 0.509), ulceration (*P* = 0.815), or Clark’s level (*P* = 0.887). Furthermore, there was no evidence of association of cyclin D1 expression with these clinicopathologic parameters, including patient age (*P* = 0.114), gender (*P* = 0.358), Breslow thickness (*P* = 0.990), ulceration (*P* = 0.198), and Clark’s level (*P* = 0.661).

## Discussion

In this study, we aimed to explore the relationship of *CCND1* copy number alteration and cyclin D1 protein expression in acral melanoma, and to determine whether anti-cyclin D1 IHC may be used as a surrogate for direct evaluation of increase in *CCND1* copy number. Our results show high-level *CCND1* copy number increase has good correlation with cyclin D1 protein expression in acral melanoma. However low-level copy number increases do not show correlation with protein expression in acral melanoma. The sensitivity (72.4%), specificity (62.5%) and positive predictive value (63.6%) of using the IHC score to predict FISH results are not good. Cyclin D1 IHC therefore cannot be used as a surrogate for direct evaluation of increase in *CCND1* copy number. Our results are consistent with the possibility *CCND1* copy number increase induce high cyclin D1 expression and promote progression in acral melanomas with high-level *CCND1* copy number increase. However, for acral melanoma with low-level *CCND1* copy number increase, copy number increase is most likely merely a result of genetic instability which occurs during tumor progression and does not induce increase in protein expression [17].

Acral melanoma is the main subtype of melanoma which affects Asian population and this melanoma subtype occurs in glabrous acral skin such as on the palms, soles, and on the nail apparatus [18]. The genomics of acral melanoma are distinct from melanoma from other cutaneous sites [19]. *CCND1* copy number increase is known to occur more commonly in acral melanomas than in melanomas in other cutaneous sites [20–24]. However the sensitivity of *CCND1* FISH for evaluation of acral melanocytic tumors is not high, and this relatively low sensitivity may result from the high heterogeneity of melanoma [25]. Both whole-genome mutation landscape and targeted genomic profiling studies demonstrate diverse oncogenic processes and genetic alterations in acral melanomas [20, 21].

In our cohort as many as 37.5% (12/32) cases without *CCND1* gene copy number increase showed high cyclin D1 protein expression, similar to the findings in a previous study [5]. In the absence of DNA copy number increase, gene overexpression may result from other mechanisms [14]. Factors other than copy number including transcriptional, post-transcriptional and translational regulation may influence cyclin D1 expression in melanoma [3, 26]. At this time we do not know the exact mechanism of high cyclin D1 expression in absence of an increased *CCND1* copy number in acral melanoma. This will be explored in our future research.

It is also noteworthy that in our cyclin D1 high-level copy number increase group, three cases (3/21, 19.0%) showed low protein expression. Cyclin D1 protein expression is regulated by a complex network, and the mechanism by which low protein expression occurs in the context of high-level increase in gene copy number is unknown. In tumors with low-level *CCND1* copy number increase, five cases (5/8, 62.5%) showed low protein expression. This indicates that low-level *CCND1* copy number increase does not lead to increase in protein expression in most cases. The copy number increase may be caused by polyploidy. When the *CCND1* copy number change is interpreted, it should be expressed in relation to one or more of the other FISH probes used.

In our cohort we found that *CCND1* copy number increase was associated with the Breslow thickness in invasive acral melanomas. That is, when invasive acral melanoma shows *CCND1* copy number increase the tumor will be thicker. This observation suggests that *CCND1* alterations may be linked to acral melanoma progression and have prognostic relevance in acral melanomas. Breslow thickness is in general the most important parameter for determining prognosis in melanoma. In our cohort some cases were consultation cases for which we failed to obtain information such as nodal status and overall survival which are more directly correlated with prognosis. We recognize this is a limitation of this study.

In summary, we found that in acral melanomas with high-level *CCND1* copy number increase IHC correlates well with FISH, while in cases with low-level *CCND1* copy number increase or no *CCND1* copy number alteration, no correlation was found. Using cyclin D1 IHC to predict *CCND1* copy number changes which can be detected by FISH is not reliable. Our findings suggested that IHC is not feasible as a surrogate for direct evaluation of *CCND1* gene copy number alteration.

## Data Availability

The data are available from the corresponding author upon reasonable request.

## Abbreviations

FISH: Fluorescence in situ hybridization
IHC: Immunohistochemistry
